# Long-term use of benzodiazepines and Z drugs: a qualitative study of patients’ and healthcare professionals’ perceptions and possible levers for change

**DOI:** 10.3399/bjgpopen18X101626

**Published:** 2019-02-20

**Authors:** Aliaksandra Mokhar, Silke Kuhn, Janine Topp, Jörg Dirmaier, Martin Härter, Uwe Verthein

**Affiliations:** 1 Scientific Associate, Department of Medical Psychology, University Medical Center Hamburg-Eppendorf, Hamburg, Germany; 2 Researcher, Department of Psychiatry and Psychotherapy, Center for Interdisciplinary Addiction Research, University Medical Center Hamburg-Eppendorf, Hamburg, Germany; 3 Scientific Associate, Institute for Health Services Research in Dermatology and Nursing, University Medical Center Hamburg-Eppendorf, Hamburg, Germany; 4 Research Group Leader, Department of Medical Psychology, University Medical Center Hamburg-Eppendorf, Hamburg, Germany; 5 Institute Director, Department of Medical Psychology, University Medical Center Hamburg-Eppendorf, Hamburg, Germany; 6 Head of Center for Interdisciplinary Addiction Research, Department of Psychiatry and Psychotherapy, Center for Interdisciplinary Addiction Research, University Medical Center Hamburg-Eppendorf, Hamburg, Germany

**Keywords:** benzodiazepines, elderly, healthcare professional, qualitative research, Z drugs, general practice

## Abstract

**Background:**

Although long-term use of benzodiazepines (BZDs) and Z drugs is associated with various side effects, they remain popular among the older population. Possible reasons for this phenomenon could be ineffective ways of transmitting information on the health risks associated with long-term use, and communication gaps between patients and healthcare professionals.

**Aim:**

The aim was to investigate the views of patients, physicians, nurses, and pharmacists regarding long-term BZD and Z drug use.

**Design & setting:**

The qualitative study design used focus group interviews with physicians, pharmacists, and nurses in Hamburg. Patient interviews were conducted in Lippstadt, Germany.

**Method:**

The interviews were audiotaped with each participant’s permission, transcribed, and thematically analysed using a software program for qualitative research (MAXQDA).

**Results:**

The data from the four focus groups consisting of 28 participants were analysed. Patients indicated lack of knowledge about risks and side effects, difficult access to alternatives, and fears of ceasing drug use without professional support. Although the physicians were reported to be cautious about prescribing BZDs and Z drugs, the psychosocial problems of older patients are often considered to be complex and treatment knowledge, experience, and resources are frequently unsatisfactory. Nurses described that when BZDs were prescribed, they did not feel it was their responsibility to evaluate their effects. Pharmacists were reported to be strongly ambivalent in informing patients about the risks, which may contradict the prescription advice provided by the physician.

**Conclusion:**

Patients, physicians, nurses, and pharmacists reported differences in the perception of long-term BZD and Z drug use. Nevertheless, all of the participants described lack of information and expressed the need for greater communication exchange.

## How this fits in

Several reasons have been identified for the associations between the long-term use of BZDs and Z drugs in the older population, and the importance of the role of communication and collaboration between patients and healthcare professionals. The results of the focus group interviews suggest that more informational exchange is needed between patients and their healthcare professionals, as well as more collaboration between different healthcare professionals.

## Introduction

The inappropriate prescribing of psychotropic drugs and polypharmacy are present in institutionalised and non-institutionalised older adults, which can cause serious side effects and might reduce patients’ quality of life.^1^ Some of the most common potentially inappropriate prescribed medications in older people are BZDs.^1^ BZDs are effective drugs for treating anxiety symptoms, as well as inducing and maintaining sleep, and muscle relaxation.^2,bib3^ The incidence of BZD prescription rates is high worldwide, and treatment duration is often inappropriately longer than the recommended maximum 8-week period.^3,bib4^ Despite the fact that these drugs are effective in the short term, long-term BZD therapy is associated with many side effects, the development of tolerance and, finally, addiction.^5^ Long-term BZD and Z drug use occurs mainly in the older population.^6^ This patient group are at particular risk of side effects because of their age-related physiological changes.^7^ Serious side effects include cognitive disturbance, an increased risk of falls and therefore hip fractures,^8–11^ hospitalisation, and increased morbidity and mortality.^12^ Continual medication use after the primary indication usually results in physical and psychological dependency,^13^ manifesting in withdrawal symptoms.

Recent research has identified several reasons for this occurrence; on the patient side, reasons for prolonged use include chronic personal stress and sleep problems, fear of recurring symptoms, lack of knowledge about risks and side effects, difficult access to alternatives, and poor motivation to cease drug use.^13–15^ Research has shown that although physicians were cautious regarding initiating BZD treatment, the psychosocial problems of patients are often considered to be complex, and knowledge is often lacking regarding managing the psychological changes associated with ageing, altered pharmacokinetics and pharmacodynamics, or using alternative strategies.^15–18^ Assessing the issues and investigating the causes of the patient's symptoms are often neglected because of a lack of resources for the management of long-term medication use by older adults.^19–21^ Pharmacists play an important role within the interprofessional healthcare team during the medical treatment of older patients. They evaluate the appropriateness, effectiveness, safety, and compliance of medications for a given patient.^22,bib23^ Other roles of the pharmacist include informing patients about the risks of using the prescribed medications, which may contradict the prescription information provided by the physician.^21,bib22^ Last but not least, nurses are involved in the healthcare management of older patients, especially in nursing homes. Evidently, older people in nursing homes often have complex illness profiles and require care and support concerning various symptoms. Nurses fulfil their duties, but they often lack responsibility regarding the medication process in relation to BZD and Z drugs.^24^

An increased emphasis on patient-centred care could address the described reasons for long-term use of BZD. International guidelines and reviews on improved medication use in general address patient-centred care dimensions and stress the importance of clinician–patient communication and/or cooperation, shared decisionmaking, and information provision.^25,26^ The explanation of the reasons for the long-term use of BZDs shows that there is missing information and a need for cooperation between healthcare professionals and patients. The aim of this study is to investigate the perspectives of physicians, nurses, pharmacists, and patients in focus groups to assess their perceptions of the reasons for long-term BZD and Z drug use and find possible solutions to the identified difficulties.

The following research questions were addressed: first, different professional groups (physicians, nurses, and pharmacists) were asked to describe long-term drug use and what they think about managing this situation from the patient’s perspective. Second, all of the participants were asked about the conditions that motivated them to seek a long-term prescription, and why it is problematical to discontinue use. Third, the participants were asked for ideas, information they need, and ways to communicate and solve the problem of long-term BZD and Z drug use.

## Method

### Study design

The qualitative study design is indicated to better understand the individual experience of the individual role of the participants, and to discuss possible solution strategies for this topic.^27^ The qualitative study design was used based on the requirements of the standard guidelines for qualitative research.^28^

### Participant recruitment and setting

A qualitative study in focus group design was conducted with patients, physicians, pharmacists, and nurses. The participants were eligible if they had been using BZDs or Z drugs for >4 weeks (patients) or if they were involved in the medical care process as doctor, pharmacist, or nurse; if they were German-speaking; and if they were physically and mentally able to take part in the focus group.

The study was performed as part of the project 'Benzodiazepines and Z drugs: concepts for risk reduction among older patients', sponsored by the Federal Ministry of Health. Physicians and pharmacists were contacted directly by the medical and pharmacist association in Hamburg. Nurses were recruited from an outpatient nursing service in Hamburg. Patients were recruited at the LWL-Klinik in Lippstadt, Germany, because of the existing cooperation in the context of the research project, in which patients with long-term BZD use are treated. All of the participants were volunteers and received financial compensation.

Four focus groups were conducted from June–August 2015. All of the applications the research team received could be included in the focus groups. There were no withdrawals. Each group comprised 6–8 participants. Focus groups with physicians and pharmacists were conducted at the University Medical Center Hamburg-Eppendorf. The focus group with nurses was held at the Martha Foundation, and the group with patients was conducted at the project associated partner LWL-Klinik. Each focus group lasted 120 minutes. Focus groups were moderated by three members of the research team.

The participants in the focus groups were informed about the research project and signed a letter of agreement.

### Data acquisition and analysis

The interview guide for the focus groups was developed based on the research questions.

- Assess views on using BZDs and Z drugs for a long time:
  - What could you say about the long-term use of BZDs and Z drugs (for example, reasons, symptoms, and knowledge about side effects)?
  - Do you think you know enough about this medication?
- Explore barriers and changing points to reduce the long-term use of BZDs and Z drugs:
  - Have you ever tried to reduce the use (as patients) or initiate the reduction (as healthcare physicians) of BZDs and Z drugs?
  - Did it work? If not, how would you explain that?
  - What do you think about the possible changing points in reducing the long-term use of BZDs and Z drugs?

The complete interviews were audio-recorded with the participants’ consent. The data were anonymised and thematically transcribed by student assistants at the University Medical Center Hamburg-Eppendorf. The content analysis was performed using MAXQDA software (version 10), which is a qualitative research software program. The MAXQDA software prepares the data for further analysis steps, in which it evaluates the transcripts to develop thematically categories. Two team members independently coded the transcripts from each focus groups. Most of the categories showed high consistency. Next, the final codes were cross-checked by a third team member. Any lack of clarity was discussed with the research team. All the information from the transcripts was used for the analysis. When more than one quotation was available for a category, only one example was selected and cited.

## Results

There were four focus groups consisting of physicians (*n* = 7), pharmacists (*n* = 6), nurses (*n* = 7), and patients (*n* = 8), as shown in Table 1 .

### Views on long-term use of BZDs and Z drugs

Prescribers apply caution in prescribing BZDs and Z drugs. Participants have reported that the long-term use of BZDs and Z drugs often starts in hospitals and its prescription is continued by GPs. The reasons for the use of these medications were sleep problems and anxiety-related symptoms producing especially an acute crisis:

*'If a patient is in an acute crisis, I often have two options: either giving him BZD, or sending him to the clinic*.' (Physician 3)

*'In the clinic, there is an even stronger tendency towards BZD, even less in line with the guideline.'* (Physician 4)

*'BZD (e.g. lorazepam) are often prescribed for anxiety, not to help patients fall asleep. I’d say patients only take it when they need it.'* (Pharmacist 6)

*'Long-term use occurs, especially in cases of mourning and when social support is lacking. Patients receive the medication during their hospital stay, notice that they slept fine and see the physician to continue the prescription.'* (Pharmacist 2)

*'Sleep disorders, anxiety, and depression are the most common causes of long-term BZD use.'* (Nurse 4)

The continued long-term prescription of BZDs and Z drugs often occurs because of problematical factors in the clinical routine, such as overcrowded waiting rooms or lack of time to speak with patients about their individual needs:

*'I have 5 minutes per patient. When one patient sits there and cries, it makes me nervous and aggressive, as I know that there are 20 other patients waiting for their doctor’s appointment outside. These are not good conditions for making a differentiated decision.'* (Physician 4)

*'If we refer the patient back to the physician, this is often perceived as an encroachment. We're not supposed to interfere in things that don't concern us.'* (Pharmacist 1)

*'We know it from our patients that they have seen the physician, waited 2 hours and were inside for 5 minutes.'* (Nurse 3)

Another reason for long-term use is that many patients are already familiar with these medications, and they directly demand prescriptions for BZDs or Z drugs without knowing about the negative aspects of the medications. In particular, pharmacists observe that patients have a careless attitude. Nurses emphasise that most patients do not demand these medications:

*'In my situation when I could not sleep anymore, I could not have taken up information about side effects anyway. I wanted a pill so I could sleep.'* (Patient 3)

*'I also realise that many patients know BZD, perhaps not the exact name but they have some knowledge, saying: “My husband has the same pill and I occasionally take some of it”.'* (Physician 2)

*'Maybe they do not even know what they are taking. They get it prescribed and they take it. I think it has less to do with memory than with the fact that they have no idea about the medication.'* (Nurse 3)

All of the professional groups agree that patients lack understanding about the likelihood of addiction regarding long-term use of BZDs and Z drugs. The drugs are fully integrated into the patients’ daily routine, awareness of potential problems is missing, and side effects such as dizziness, unsteadiness while walking, or depression are not associated with the medications. The patients themselves claimed that they did not feel addicted, although they were aware that consistent intake was present, and the physicians claimed to clearly emphasise the side effects:

*'I remember being fixed on a single pill of lexotanil for years: no more, no less. In the evening exactly 6 mg bromazepam* […] *and I did not think about dependency until I stopped taking the drug and noticed these withdrawal symptoms. So, I was literally trapped.'* (Patient 5)

*'Again and again, I experience that patients do not have a feeling for what they are taking.'* (Physician 6)

*'Patients do not have the impetus to say: "I want to get away from it". The medication is integrated in their everyday lives. Patients have no problem awareness, and nobody addresses the problem, especially when they live alone.'* (Pharmacist 3)

Nevertheless, the pharmacist tends to take a critical view of the physician informing the patient about all types of side effects (including the dangers of addiction):

*'As pharmacists, we have an awe of medicines and we do not experience this awe in the everyday life of the patients and the physicians … For them, it is self-evident. When I am hungry, I eat a piece of bread. When I have a headache, I take a pill. When I cannot sleep, I take a pill. That’s it.'* (Pharmacist 1)

Nurses commented that many patients could not say why they had received their medication after their appointment with the physician, and said that patients had not been informed because of the brief consultation time:

*'* […] *especially the older ladies and gentlemen, they are happy if they had seen the physician and left with a prescription of a new medication. And once they are asked what the physician explained to them, they say it was too fast and they had no time to ask questions.'* (Nurse 4)

### Barriers and changing points to reduce the long-term use of BZDs and Z drugs

Nearly all of the interviewed patients had the experience of receiving BZDs over a long to very long period (ranging from a number of weeks to many years) without any difficulties and then, suddenly and inexplicably, they were denied the prescription or they were urged to discontinue the medication. There were no preparatory discussions or jointly made decisions, according to them:

*'I have taken the medication for years and subsequently increased the dosage* [because of husband’s care and death]*. One day, the physician, who had been prescribing the medications, said, “I think the medication needs to be withdrawn”.'* (Patient 4)

While physicians and some nurses tend not to initiate medication discontinuation among the oldest patients nor discuss the dangers of addiction, pharmacists believe discontinuing the drugs is beneficial at any stage of life. Pharmacists also report that GPs often abruptly stop prescribing, without suggesting a more gradual discontinuation process. Based on this experience, they recommend and motivate their customers to contact a psychiatrist, who can initiate and support a qualified stepwise reduction:

*'If patients are aware of the problem, we encourage them to find another physician* [psychiatrist] *who can competently advise discontinuation of drug use.'* (Pharmacist 2)

*'I have a patient *[female, aged 85 years]*, I have been prescribing drugs to for years and I will continue prescribing BZD to her for the rest of her life. I do not see the point in discontinuing.'*(Physician 6)

*'Discontinuation rarely happens and is very difficult particularly in older people. Patients start asking: “Where is half my pill for the night?” or they tell me: “I cannot sleep without it.” There is no possibility of discontinuing the medication because they insist on this pill, whether they really need it or not.'* (Nurse 1)

Alternative treatments were not discussed, and in one case, they were denied. Pharmacists believe that if prescribers with further experience and knowledge of, for instance, homeopathy or palliative medicine manage their patients more thoroughly, they would be more likely to oversee attempts at discontinuation of drug use:

*'Being a physician includes addressing alternative treatment options. I think that this is a problem for many physicians.'* (Pharmacist 2)

## Discussion

### Summary

In the four focus groups with patients, physicians, pharmacists, and nurses, the primary reasons for prescribing BZDs and Z drugs were identified. These reasons were often sleep problems, anxiety symptoms, and individual crises, and the initial drug use is often in an acute hospital setting. The reasons for transitioning to long-term drug use are varied. Patients are often not informed of the potential risks and side effects when they initially receive the drug. Often patients do not know who to contact when the drug use exceeds the expiration date, nor do they know with whom to discuss medical issues when symptoms occur. The majority of the patients do not feel that using this medication is a problem. Physicians see the responsibility for the use of BZDs as in the patient's hands, and vice versa. Furthermore, there is often a lack of resources, time, or specific knowledge regarding how to address sleep- or anxiety-related symptoms in older patients. Noticing reckless drug prescriptions and intake behaviours, pharmacists often inform patients and motivate them to discontinue the medication. Nonetheless, pharmacists are hesitant to contact the physician. Nurses noticing the problematic BZD and Z drug use often feel unsure, and lack competency and knowledge to inform patients or initiate discontinuation of the medication.

To the authors' knowledge, this qualitative study is the first of its kind that looks at the perceptions of patients, as well as different healthcare professionals, on long-term use or prescription of BZDs and Z drugs. As has been found in previous studies exploring BZD use from single perspectives, the authors of the present study conclude that long-term use is an ongoing problem particularly in older patients.^1,6^ Although physicians seem to be more cautious in prescribing medications, further strategies need to be developed to tackle inappropriate long-term use. Therefore, the following issues need to be addressed: physicians do not know of appropriate treatment alternatives; patients have insufficient knowledge on health risks associated with long-term intake of BZDs; and different professions collaborate insufficiently.

Recommendations for the improvement of the care process can be derived from the reported reasons for long-term use and barriers to the guideline-compliant prescription. Recommendations are based on the principles of patient-centred care, which is an approach in health care that focuses on the individual needs and values of the patient, and aims to improve the general quality of the care process.^29^ The integrated model of patient-centredness, as described by Scholl *et al* ^30^ includes 15 dimensions of patient-centred care, many of which could be referred to in order to improve BZD use and prescription in the older population.

The results from the focus groups support the view that present difficulties in health care may be solved by changing the perceptions of one’s role in the care process. For example, for physicians, there is an evident need for improved clinician–patient communication, which is essential for patient-centred care. It is necessary to actively involve patients in the treatment process.^31,32^

Patients often ask for comprehensive information and discussions with their physician; therefore, a more active role in treatment and support for receiving self-management is needed.^33^ Self-management education is particularly important for older patients, to enable them to manage their symptoms.^34^ None of the patients interviewed were able to recall such a discussion, while all believing that comprehensive clinician–patient communication would have prevented inappropriate use of BZDs. Many studies have linked patient information with improved heath and increased health-related quality of life.^35–37^ Joint consideration about the best course of treatment should form the primary focus, and several evidence-based communication methods could facilitate that process.^35^

Furthermore, communication between different healthcare professionals is needed to ensure coordination and continuity of care, which is another facilitator for patient-centred care. Active interaction between all stakeholders, open communication, and a consensual clear line of treatment is recommended in order to best meet the need of the individual patient.^26,27^ This includes the transition between inpatient and outpatient care, as well as the coordination of care in the outpatient setting.

The results from the focus groups indicate that pharmacists and nurses are engaged closely with patients. The close relationship between pharmacist and patient is, so far, underutilised for the optimisation of the prescription. Pharmaceutical expertise rarely shapes and impacts a physician’s decisionmaking. Pharmacists express uneasiness about contacting physicians and discussing problematic prescriptions. They also worry that informing the patients about prescription problems might interfere with the physician’s advice and might cause the patient–physician relationship to become distant or strained, thus creating potential conflict. Active exchange between both groups should allow the pharmacist to call the physician in cases of problematic or complex prescriptions. Arranging a time to discuss specific questions may be an option, in which the patient’s health will be the primary focus. This change of action and thinking could result in shared responsibility, improved coordination, and continuity of care.

The results from the focus groups also suggest room for improvement in nursing. Nurses are willing to assume more responsibility and play a more active part in medicinal treatment, serving as a link between physicians and patients. While case conferences may be created by recapping the documentation of a complex course, professional exchange may occur between the person in charge of the nursing team and a physician colleague. The subsequent responsibility of passing on conference results lies with the respective disciplines. These steps create benefits for all parties concerned. Physicians can transfer parts of their expertise to nursing, thereby sharing some of the responsibility while retaining a close watch. Nurses, meanwhile, gain knowledge and skills allowing them to handle specific medicinal problems, and they assume increased responsibility for the patient. They may reflect about the benefits and limitations of medicinal treatment, and they can support the patient in overcoming the difficulties at hand. Improved communication and cooperation across all parties would help to ensure successful treatment of older patients; indeed, the importance of communication and cooperation form part of the international guidelines for improving the quality of healthcare systems.^1^

### Strengths and limitations

The present study has several strengths. First, four different and very important perspectives on the problem of long-term use of BZDs and Z drugs were presented. Second, the problems were identified and various practical options were considered with the agreement and support for all interviewed parties. Third, the sample size of each focus group was appropriate according to the method.^38,39^

There are several limitations to the study. First, the recruitment of the participants proved to be difficult. It is, therefore, assumed that the participants took part in the study because of the financial incentive. The research team could not completely control the number of or characteristics of the invited participants, and there is a likelihood of selection bias. The sample was a convenience sample recruited through several pathways. The authors only captured the sample size and sex of the healthcare professionals. Second, there is a limitation regarding the sex of the sample size because it consisted of more female participants than male. Third, in the focus group, the issues of reducing the long-term use of BZD and Z drugs were not discussed in detail due to time constraints. Future research is warranted to produce an additional perspective on this matter. Fourth, the focus was on BZD and Z drug use in older age and important issues, such as polypharmacy, physical illness, or social situation, were not discussed. Fifth, the bias of the personality of the moderator was minimised by providing a structured steps protocol of questions and also by discussing any abnormalities regarding the interviews. Nevertheless, moderator bias still existed.

### Comparison with existing literature

Training to improve communication and collaboration with other specialists in the field of deprescribing BZDs and Z drugs in the older population is needed.^23^ Furthermore, previous studies have produced sophisticated and multifaceted concepts that address cooperation among the various parties, improve educational activities, and communication exchange with patients.^24,40^ Collaborative team-based models can improve safe medication use and treat the symptoms.^41^ Dollman and colleagues' multistrategic approach highlights the benefits of providing medication reduction care to older people, showing a significant reduction in BZD use while successfully promoting new non-medical alternatives.^42^ Those concepts could be transferred and integrated into ambulatory care in Germany.

Assisting and supporting patients in drug withdrawal is possible in several ways. For example, while some patients may seem to require a lot of effort, several studies have shown that patient-centred care, combined with a specific method for drug discontinuation, can reduce and stop BZD use.^21,43^

### Implications for research and practice

Those involved in the healthcare system, namely, nurses, physicians, pharmacists, and patients, play increasingly interdependent roles. Problems arise every day that do not have easy or singular solutions. Leaders who merely give directions and expect them to be followed will not succeed in this environment. What is needed is a style of leadership that involves working with others as full partners in a context of mutual respect and collaboration. For this, new evidence-based approaches to better communication and collaboration between the different healthcare professionals are needed. A systematic review is needed to synthesise knowledge about all existing interventions that focus on the long-term use of BZDs in older people and combined approaches for the stakeholders. Physicians, pharmacists, and nurses must work together to break down the walls of hierarchical silos and hold each other accountable for improving quality, and decreasing preventable adverse events and medication errors. Future research should pay more attention to the sample characteristics; a more comprehensive description is needed of the individual characteristics of the healthcare professionals (such as age and years working). All of these players must display the ability to adapt to the continually evolving dynamics of the healthcare system. There is a need for more scientific research to better understand which strategies are most effective and could be implemented in the daily clinical routine.

**Table 1.** Description of sample size

Focus group	Sample size, *n*	Male, *n*	Female, *n*	Participant characteristics
1 Patients	8	3	5	P1: late 40s, male patient, 22 years BZD-dependent P2: 24 year old male patient, 3 years of BZD use P3: 75 year old female patient, 29 years of BZD use, 2.5 years without BZD P4: 85 year old female patient, 40 years of BZD use, 3 years without BZD P5: 58 year old male patient, 20 years of BZD and opiate use with massive dose increase, 6.5 years without BZD and opiates P6: mid-50s female patient, 30 years of use of opiates and occasional BZD, detoxified for the last 3 days P7: late 30s, female pain patient, BZD (if needed) P8: late 40s, female pain patient, BZD (if needed)
2 Physicians	7	2	5	Working area: 3 × own practice 1 × practice and hospital 2 × psychiatric hospital, institute outpatient clinic 1 × psychiatrist (parental leave)
3 Pharmacists	6	0	6	All of them reported years of experience in pharmacies throughout Hamburg
4 Nurses	7	0	7	Working area: 2 × inpatient care 5 × outpatient care, including one nursing service and one task line

## References

[1] Ćurković M, Dodig-Ćurković K, Erić AP (2016). Psychotropic medications in older adults: a review. Psychiatr Danub.

[2] Mehdi T (2012). Benzodiazepines revisited. British Journal of Medical Practitioners.

[3] Gallagher H (2013). Addressing the issue of chronic, inappropriate benzodiazepine use: how can pharmacists play a role?. Pharmacy.

[4] Holzbach R (2010). [Benzodiazepine long-term use and dependence] *Benzodiazepin-Langzeitgebrauch und -abhängigkeit* (in German). Fortschritte der Neurologie-Psychiatrie.

[5] Smink BE, Egberts AC, Lusthof KJ (2010). The relationship between benzodiazepine use and traffic accidents: A systematic literature review. CNS drugs.

[6] Olfson M, King M, Schoenbaum M (2015). Benzodiazepine use in the United States. JAMA Psychiatry.

[7] Wolter DK (2008). [Main topic: sleep aids and sedatives in old age] *Themenschwerpunkt: Schlaf- und Beruhigungsmittel im Alter* (in German). Zeitschrift für klinische Psychologie, Psychopathologie und Psychotherapie.

[8] Ray WA, Griffin MR, Downey W (1989). Benzodiazepines of long and short elimination half-life and the risk of hip fracture. JAMA.

[9] Rummans TA, Davis LJ, Morse RM (1993). Learning and memory impairment in older, detoxified, benzodiazepine-dependent patients. Mayo Clin Proc.

[10] Pat McAndrews M, Weiss RT, Sandor P (2003). Cognitive effects of long-term benzodiazepine use in older adults. Hum Psychopharmacol.

[11] Cumming RG, Le Couteur DG (2003). Benzodiazepines and risk of hip fractures in older people. A review of the evidence. CNS Drugs.

[12] Clyne B, Bradley MC, Hughes CM (2013). Addressing potentially inappropriate prescribing in older patients: development and pilot study of an intervention in primary care (the OPTI-SCRIPT study). BMC Health Serv Res.

[13] Cook JM, Biyanova T, Masci C (2007). Older patient perspectives on long-term anxiolytic benzodiazepine use and discontinuation: a qualitative study. J Gen Intern Med.

[14] Anthierens S, Habraken H, Petrovic M (2007). First benzodiazepine prescriptions: qualitative study of patients' perspectives. Can Fam Physician.

[15] Anthierens S, Habraken H, Petrovic M (2007). The lesser evil? Initiating a benzodiazepine prescription in general practice. Scand J Prim Health Care.

[16] Parr JM, Kavanagh DJ, Young RM (2006). Views of general practitioners and benzodiazepine users on benzodiazepines: a qualitative analysis. Soc Sci Med.

[17] Perodeau G, Grenon É, Grenier S (2016). Systemic model of chronic benzodiazepine use among mature adults. Aging Ment Health.

[18] Turnheim K (2004). Drug therapy in the elderly. Exp Gerontol.

[19] Brett J, Murnion B (2015). Management of benzodiazepine misuse and dependence. Aust Prescr.

[20] Tjagvad C, Clausen T, Handal M (2016). Benzodiazepine prescription for patients in treatment for drug use disorders: a nationwide cohort study in Denmark, 2000–2010. BMC Psychiatry.

[21] Martin P, Tamblyn R, Ahmed S (2013). An educational intervention to reduce the use of potentially inappropriate medications among older adults (EMPOWER study): protocol for a cluster randomized trial. TRIALS.

[22] Farrell B, Shamji S, Dalton D (2014). Managing chronic diseases in the frail elderly: More than just adhering to clinical guidelines. Can Pharm J (Ott).

[23] Schuling J, Gebben H, Veehof LJ (2012). Deprescribing medication in very elderly patients with multimorbidity: the view of Dutch GPs. A qualitative study. BMC Fam Pract.

[24] Forsetlund L, Eike MC, Gjerberg E (2011). Effect of interventions to reduce potentially inappropriate use of drugs in nursing homes: a systematic review of randomised controlled trials. BMC Geriatr.

[25] Légaré F, Stacey D, Turcotte S (2014). Interventions for improving the adoption of shared decision making by healthcare professionals. Cochrane Database Syst Rev.

[26] Royal Australian College of General Practitioners, Newman R (2015). Prescribing drugs of dependence in general practice. Part B Benzodiazepines. https://www.racgp.org.au/FSDEDEV/media/documents/Clinical%20Resources/Guidelines/Drugs%20of%20dependence/Prescribing-drugs-of-dependence-in-general-practice-Part-B-Benzodiazepines.pdf.

[27] Wilkinson S (1998). Focus group methodology: a review. Int J Soc Res Methodol.

[28] Tong A, Sainsbury P, Craig J (2007). Consolidated criteria for reporting qualitative research (COREQ): a 32-item checklist for interviews and focus groups. Int J Qual Health Care.

[29] Institute of Medicine (US) Committee on Quality of Health Care in America (2001). Crossing the quality chasm: a new health system for the 21st century.

[30] Scholl I, Zill JM, Härter M (2014). An integrative model of patient-centeredness — a systematic review and concept analysis. PLoS One.

[31] Loh A, Simon D, Kriston L (2007). [Patient involvement in medical decisions] *Patientenbeteiligung bei medizinischen Entscheidungen* (in German). Deutsches Ärzteblatt.

[32] Joosten EA, DeFuentes-Merillas L, de Weert GH (2008). Systematic review of the effects of shared decision-making on patient satisfaction, treatment adherence and health status. Psychother Psychosom.

[33] de Silva D (2011). Evidence: helping people help themselves. A review of the evidence considering whether it is worthwhile to support self-management. https://www.health.org.uk/sites/default/files/HelpingPeopleHelpThemselves.pdf.

[34] Bodenheimer T, Lorig K, Holman H (2002). Patient self-management of chronic disease in primary care. JAMA.

[35] Mugunthan K, McGuire T, Glasziou P (2011). Minimal interventions to decrease long-term use of benzodiazepines in primary care: a systematic review and meta-analysis. Br J Gen Pract.

[36] Rao JK, Anderson LA, Inui TS (2007). Communication interventions make a difference in conversations between physicians and patients: a systematic review of the evidence. Med Care.

[37] Farmer AP, Légaré F, Turcot L (2008). Printed educational materials: effects on professional practice and health care outcomes. Cochrane Database Syst Rev.

[38] Marshall B, Cardon P, Poddar A (2013). Does Sample Size Matter in Qualitative Research?: A Review of Qualitative Interviews in is Research. Journal of Computer Information Systems.

[39] Sandelowski M (1995). Sample size in qualitative research. Res Nurs Health.

[40] Grimshaw JM, Shirran L, Thomas R (2001). Changing provider behavior: an overview of systematic reviews of interventions. Med care.

[41] Furbish SML, Kroehl ME, Loeb DF (2017). A pharmacist-physician collaboration to optimize benzodiazepine use for anxiety and sleep symptom control in primary care. J Pharm Pract.

[42] Dollman WB, Leblanc VT, Stevens L (2005). Achieving a sustained reduction in benzodiazepine use through implementation of an area-wide multi-strategic approach. J Clin Pharm Ther.

[43] Tannenbaum C, Martin P, Tamblyn R (2014). Reduction of inappropriate benzodiazepine prescriptions among older adults through direct patient education: the EMPOWER cluster randomized trial. JAMA Intern Med.

